# Supplementary material for: Prediction of Response and Survival Following Treatment with Azacitidine for Relapse of Acute Myeloid Leukemia and Myelodysplastic Syndromes after Allogeneic Hematopoietic Stem Cell Transplantation
Source: Cancers (Basel). 2020 Aug 12;12(8):2255. doi: 10.3390/cancers12082255 (PMC7464210; doi:10.3390/cancers12082255)
Supplement: Supplementary file 1 [file cancers-12-02255-s001.pdf]

Supplemental Material

Supplemental Figure 1. Landmarkanalysis regarding OS of patients who received DLI compared to those who did not. As the median time to application of first DLI were 55 days, this time point was chosen as a landmark for survival analyses.

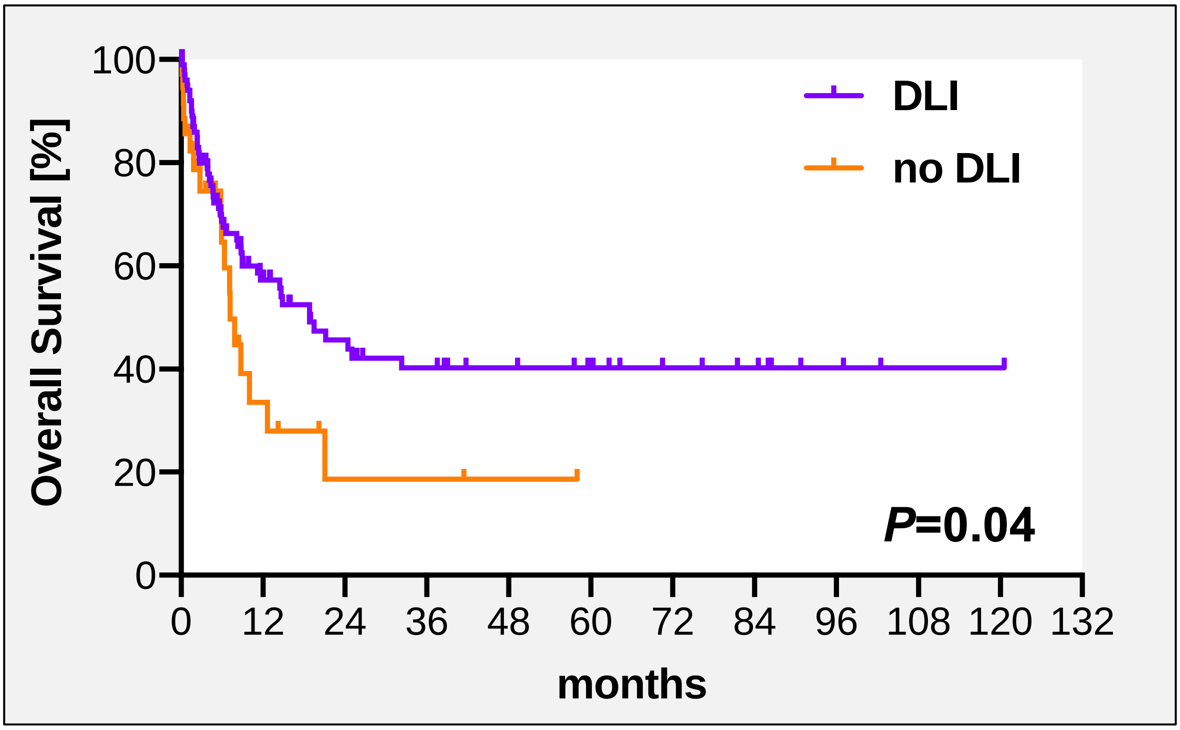

Supplement Figure 2. Overall survival (OS) after treatment with Azacitidine ± DLI in 77 patients with AML and MDS treated with Azacitidine/DLI for hematologic relapse after allo-HSCT according to the Aza Relapse Prognostic Score (11). The time between transplant and relapse was assigned with 2 points if less than six months, 1 if 6-12 months and 0 if greater than 12 months. A blast percentage greater than the median (20%) at relapse was assigned 1 point, while a blast percentage below 20% was assigned with 0 points.

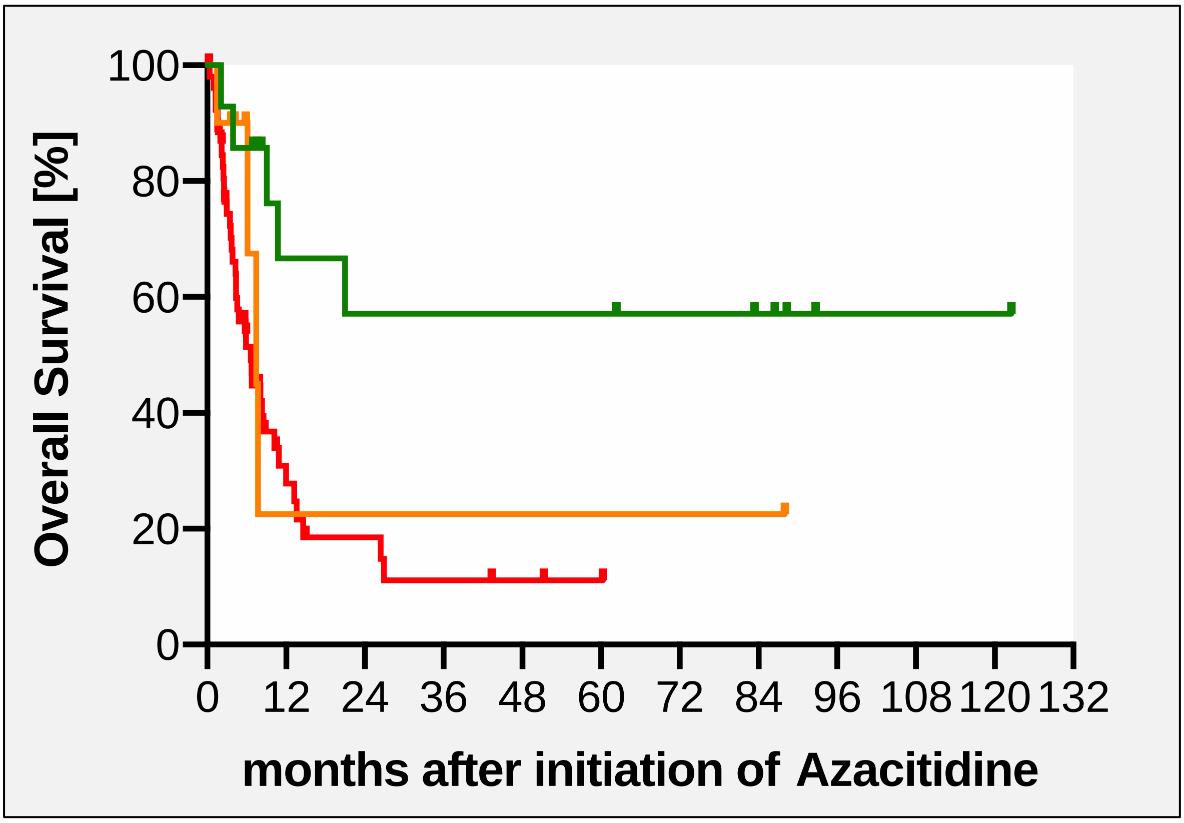

group 1 vs. group 2, *P* = 0.13

group 2 vs. group 3, *P* = 0.36

Supplemental Table 1. Conditioning regimen

	All patients	upfront	CTX	HMA
**No.**	147	43	90	14
**Conditioning, n (%)**				
FLAMSA-Melphalan	75 (51)	28 (65)	41 (46)	6 (43)
FLAMSA-Treosulfan	9 (6)	2 (5)	7 (8)	-
Fludarabine-Treosulfan	25 (17)	5 (12)	14 (16)	6 (43)
Fludarabine-Busulfan	15 (10)	6 (14)	9 (10)	-
Fludarabine-TBI	2 (1)	1 (2)	1 (1)	-
Busulfan-Cyclophosphamid	5 (3)	-	5 (6)	-
Other	15 (10)	1 (2)	12 (13)	2 (14)
Missing information	1 (1)	-	1 (1)	-

Information about pretransplant strategy missing in 4 patients.

Supplement Table 2: Impact of Clinical Parameters on Response and Outcome after Treatment with Azacitidine for post-transplant sAML# and MDS relapse – Univariate and Multivariate Analysis (n=66)

Variable	Overall Survival			Response		
	2-yr OS after commencement of Aza (%)	*P*		CR Rate after commencement of Aza (%)	*P*	
		Univariate	Multivariate		Univariate	Multivariate
**Age**						
≥ 60 yrs	34.4 ± 9	0.1811	-	33	0.5102	-
< 60 yrs	42.7 ± 9			41		
**Gender**						
Female	30.4 ± 10	0.2252	-	26	0.0516	0.003
Male	44.8 ± 8			47		
**Karyotype**						
Abnormal	32.6 ± 8	0.1513	-	32	0.4722	-
Normal	56.2 ± 12			41		
**Karyotype**						
Complex	26 ± 9	0.1541	0.021	31	0.3692	-
Not complex	47.3 ± 9			41		
**Cytogenetic risk***						
Poor/very poor	26.6 ± 9	0.1338	-	33	0.3843	-
Very low/low/int	48.1 ± 9			43		
**Disease status at transplant**						
No CR	44.6 ± 8	0.4959	-	41	0.5965	-
CR	33.3 ± 15			32		
**Pretransplant strategy**						
Treated	25.3 ± 6	0.0004	0.009	28	0.0130	0.006
Upfront	63.5 ± 10			56		
**Donor**						
Unrelated	44.6 ± 7	0.8631	-	39	0.9999	-
Related	18.3 ± 16			35		
**HLA-Match**			-			

Mismatched	37.2 ± 11	0.6139		31	0.4748	-
Matched	38.9 ± 8			42		
**Conditioning**[‖]						
RIC	30.7 ± 8	0.2975	-	32	0.1185	-
Standard dose	48.7 ± 10			50		
**Type of relapse**						
Hematologic	33.9 ± 8	0.0232	ns	27	0.0086	0.002
Molecular	50.6 ± 12			56		
**Time until relapse**						
<6 months	32.4 ± 9	0.0106	ns	46	0.1333	-
≥6 months	52.7 ± 10			54		
**BM blasts at relapse**						
>8% (median)	33 ± 10	0.0012	0.001	24	0.0070	-
≤8%	47.3 ± 9			54		

[#]besides MDS only patients with sAML with 20-29% BM blasts (formerly RAEB-T) were included

*according to Greenberg et al. (20)

[‖]according to Bacigalupo et al. (21)

- indicates that the respective parameter was not included into multivariate model

AML, acute myeloid leukemia; Aza, Azacitidine; BM, bone marrow; CR, complete remission; HLA, human leukocyte antigen; int, intermediate; MDS, myelodysplastic syndrome; ns, no statistical significance; RIC, reduced intensity conditioning; yr, years; yrs, years

Supplement Table 3: Two-year overall survival and response rate of 77 patients with AML and MDS treated with Azacitidine ± DLI for hematologic relapse after allo-HSCT according to the Aza Relapse Prognostic Score (11)

Risk Score/ Group	Response Rate (CR) after Azacitidine	2-yr OS Rate after Azacitidine [± SEM]
**1 (n=14)**	57%	57% ± 15%
**2 (n=10)**	40%	23% ± 20%
**3 (n=53)**	18%	19% ± 6%
	P = 0.01	P = 0.01

Allo-HSCT, allogeneic hematopoietic stem cell transplantation; AML, acute myeloid leukemia; BM, bone marrow; CR, complete remission; DLI, donor lymphocyte infusion; MDS, myelodysplastic syndrome; OS, overall survival; sAML, secondary AML; SEM, standard error of the mean; yr, year

The time between transplant and relapse was assigned with 2 points if less than six months, 1 if 6-12 months and 0 if greater than 12 months. A blast percentage greater than the median (20%) at relapse was assigned 1 point, while a blast percentage below 20% was assigned with 0 points.
